# Clinical and Laboratory Characteristics of Dengue-*Orientia tsutsugamushi* co-Infection from a Tertiary Care Center in South India

**DOI:** 10.4084/MJHID.2016.028

**Published:** 2016-06-16

**Authors:** Aneesh Basheer, Nayyar Iqbal, Sudhagar Mookkappan, Patricia Anitha, Shashikala Nair, Reba Kanungo, Ravichandran Kandasamy

**Affiliations:** 1 Department of General Medicine, Pondicherry Institute of Medical Sciences, Ganapathichettikulam, Kalapet, Pondicherry – 605014; 2 Department of Microbiology, Pondicherry Institute of Medical Sciences, Ganapathichettikulam, Kalapet, Pondicherry – 605014; 3 Department of Biostatistics, Pondicherry Institute of Medical Sciences, Ganapathichettikulam, Kalapet, Pondicherry – 605014

## Abstract

**Background:**

Concurrent infection with multiple pathogens is common in tropics, posing diagnostic and treatment challenges. Although co-infections of dengue, malaria, leptospirosis and typhoid in various combinations have been described, data on dengue and scrub typhus co-infection is distinctly limited.

**Methodology:**

This study was a retrospective analysis of dengue and scrub typhus co-infection diagnosed between January 2010 and July 2014 at a tertiary care center. Clinical and laboratory features of these cases were compared with age and gender-matched patients with isolated dengue fever and isolated scrub typhus. Positive test for dengue non-structural 1 (NS1) antigen was considered diagnostic of dengue whereas scrub typhus was diagnosed by IgM scrub antibodies demonstrated by ELISA.

**Results:**

There were 6 cases of dengue-scrub co-infection during the review period which fitted clinical and laboratory profile with a mean age of 42.5 years. Fever, headache, and arthralgia were common. Normal hemoglobin, significant thrombocytopenia, transaminitis, and hypoalbuminemia were identified in these patients. Compared to patients with isolated dengue, those with co-infection had higher pulse rate, lower systolic blood pressure, normal leucocyte counts, higher levels of liver enzymes, greater prolongation of partial thromboplastin time (aPTT) and lower serum albumin. Co-infection was characterized by a lower nadir platelet count compared to scrub typhus, and lesser time to nadir platelet count and longer duration of hospital stay compared to either isolated dengue or scrub typhus.

**Conclusion:**

Dengue-scrub typhus co-infection may be under-diagnosed in tropics, particularly confounded during dengue epidemics. Normal leukocyte counts, early drop in platelets and hypoalbuminemia in dengue patients could be clues to concurrent scrub typhus infection. Prompt recognition and treatment of scrub typhus in such cases may reduce unnecessary hospital stay and cost.

## Introduction

Dengue, a mosquito-borne viral infection and scrub typhus, a mite-borne rickettsial infection are common causes of acute febrile illness in the tropics. During the seasonal increase in dengue cases, concomitant scrub typhus infection in endemic areas can cause a diagnostic dilemma. Both diseases have several clinical and laboratory features in common, including rash, thrombocytopenia, and hepatic dysfunction. However, concurrent infection with both pathogens is exceedingly rare, primarily due to the different vectors involved. We aim to describe the clinical and laboratory features of dengue-scrub typhus co-infection from a tertiary hospital along the southern coast of India.

## Methods

We reviewed medical records from January 2010 to July 2014 for concomitant diagnosis of dengue fever and scrub typhus. Fourteen records were identified with this combined diagnosis. However, in 6 of them, the diagnosis of scrub typhus was based on clinical features and the presence of an eschar. ELISA for IgM antibodies to *Orientia tsutsugamushi* (*O.tsutsugamushi*) was not done in these cases. Of the remaining 8 cases, 2 lacked complete clinical and laboratory details. Finally, 6 cases of dengue-scrub typhus co-infection fitted the criteria of inclusion for the analysis. In all these cases, dengue fever had been confirmed by a positive test for Non-structural 1 (NS1) antigen (J Mitra & Co, New Delhi) with or without IgM antibodies to the virus. Scrub typhus had been diagnosed based on compatible clinico-laboratory features and a positive test for IgM antibodies to *Orientia tsutsugamushi* by ELISA (Pan Bio-USA). The cut off had been calculated based on the standard curve for the kit determined by the Receiver Operative Characteristic (ROC) curves obtained from testing sera of three groups of individuals. Testing was done on 100 serum samples each, of the known scrub typhus positive group, sera from patients with other febrile illnesses (malaria, enteric fever, dengue and urinary tract infection) and sera from normal healthy volunteers. The results thus obtained were used to determine the cutoff to detect positive and negative results. The clinical protocol was fever, with or without an eschar, respiratory involvement in the form of pneumonitis and a strong history of outdoor activities in the recent past.

Eighteen age and gender-matched patients with a diagnosis of isolated dengue fever were also analyzed. Clinical characteristics including duration of fever, headache, presence of rash, arthralgia and organomegaly were noted. Laboratory findings such as blood counts, liver functions, renal functions and coagulation profile were also determined. Serial blood counts were available in all cases till discharge and these were used to identify lowest platelet count and time to attainment of nadir count. Other investigation results were collected based on tests ordered at or within 24 hours of admission. Similarly, clinical and laboratory characteristics of another eighteen age and gender matched individuals diagnosed with isolated scrub typhus were also determined. These were compared with patients having dengue-scrub typhus co-infection.

### Statistical analysis

Descriptive methods were used to present data related to clinical features of patients. One way ANOVA was done to compare means of the three groups for normally distributed variables and Tukey HSD method for multiple comparisons. For non-normally distributed variables the medians were compared using Kruskal-Wallis test with post hoc test for multiple comparisons. Chi-square test was performed to determine the association between categorical variables. All tests were two-sided. Differences were considered significant if the p-value was less than 0.05.

### Ethics statement

The Institute Ethics Committee (IEC) of the Pondicherry Institute of Medical Sciences approved and permitted waiver of consent for the study as this was a retrospective data analysis and did not directly involve patients. All data analyzed were anonymized.

## Results

During the period between January 2010 and July 2014, there were 6 cases with a confirmed diagnosis of dengue and scrub typhus co-infection. The mean age of the patients was 42.5 years, and 67% were males. Headache was the most common symptom apart from fever. Fever was present in all patients at admission and median duration of fever at the time of admission was 5.5 days. Arthralgia was seen in a significant number of patients (50%). Rash was observed in 2 (33%) patients, and it appeared as a diffuse blanching erythematous rash with no documentation of petechiae or purpura. Eschar was noted in 33% patients (one in the groin and other in the left axilla). Hepatosplenomegaly was uncommon (Table 1).

The mean hemoglobin of the patients was 10.6 g/dL. Only one patient had elevated total leukocyte counts, whereas all others had normal counts or leucopenia. The median lowest (nadir) platelet count was 21.7 x 10^9^ cells/L. Liver functions revealed transaminitis in all patients, and elevation of AST and ALT was more than three times the upper limit of normal in 83% of patients. AST/ALT ratio was more than one in all patients. Mild hypoalbuminemia was observed (mean serum albumin − 3g/dl).

### Comparison of clinical and laboratory parameters

We compared the clinical and laboratory characteristics of patients with dengue-scrub co-infection, with the isolated forms of dengue fever, and scrub typhus. Among the clinical variables, patients with co-infection had a significantly higher pulse rate compared to those with only dengue fever (97.0 ± 7.6 vs 81.2 ± 11.9; p = 0.01). Median systolic blood pressure was lower in patients with co-infection [99 (94 – 107) vs 118 (110 – 130) mm Hg; p = 0.01].

Patients with co-infection had normal mean leucocyte counts (8.8×10^9^ cells/L) compared to leucopenia (3.9×10^9^/L; p < 0.001) in dengue fever and mild leukocytosis in scrub typhus (11.6×10^9^/L; p = 0.01). Aspartate aminotransferase (AST) and alanine aminotransferase (ALT) were considerably higher in the co-infection group compared to isolated dengue fever and isolated scrub typhus (Table 2). The serum albumin levels were significantly lower in co-infection as compared to isolated dengue (3.0 vs 3.9 g/dl; p < 0.001) but not with isolated scrub typhus (3.0 vs 2.9 g/dl; p = 0.95).

Hemoconcentration was conspicuous in the isolated dengue group whereas that with co-infection had lower mean hemoglobin. The drop in platelet counts during illness was most marked in co-infection and was significantly lower compared to those with isolated scrub typhus (Table 2). The nadir platelet count occurred much earlier in patients with co-infection compared to those with isolated dengue and isolated scrub typhus (Table 3).

All six patients with co-infection received antibiotics; four of them were prescribed oral doxycycline and the others azithromycin. Three patients were started on antibiotics before the results of IgM for scrub were available based on clinical suspicion, whereas the other three received antibiotics after laboratory confirmation of scrub typhus co-infection. All of them responded within 48 hours of antibiotic initiation with temperature touching baseline.

## Discussion

Scrub typhus is a rickettsia infection that is being increasingly diagnosed in the tropics. In fact, it is estimated that scrub typhus accounts for 40–50% of cases of undiagnosed acute febrile illnesses.^1^ On the other hand, dengue fever has become endemic in most parts of Southeast Asia with outbreaks occurring with clockwork precision every year in and around the rainy seasons. Of late the seasonal variation of occurrence of dengue is slowly getting obliterated, with isolated cases being reported throughout the year. It is not uncommon to detect concurrent infections with multiple pathogens in the same individual in developing countries, especially during periods of the outbreak. Dengue co-infection with leptospirosis and malaria has been described in the literature.^2^ Recent studies also report a high incidence of concurrent infection with dengue and *Salmonella* Typhi.^3^ However, only one study from North India and a case report from this institute has been reported, on dengue-scrub typhus co-infection to the best of our knowledge.^4^,^5^ Clinical and laboratory features of both the diseases have been extensively studied and described separately. We describe the clinical and laboratory features of patients diagnosed with this rare combination of infections and compare with those of patients with isolated dengue fever and isolated scrub typhus.

Duration of fever, mean age of presentation and male to female ratio in our study were similar to the study by Ahmed et al.^4^ Abdominal pain was a common clinical manifestation in the above study, whereas headache was the common clinical feature apart from fever in our study. Patients with co-infection had symptoms indistinguishable from isolated dengue fever. Eschar, the pathognomonic feature of scrub typhus was found in only one-third of our patients. Comparison of co-infection cases with isolated dengue revealed certain interesting observations. A higher pulse rate and lower systolic blood pressure were findings that suggested co-infection whereas other clinical findings differed little between patients with co-infection and those with dengue fever. In contrast to patients with dengue fever, those with co-infection were likely to have normal leukocyte counts. Interestingly, although the nadir platelet count did not differ, time taken for the maximum drop in platelet count differed significantly between these two groups of patients, with nadir attained earlier in those with co-infection. In contrast, co-infection patients had both lower nadir platelet counts and earlier nadir compared to patients with isolated scrub typhus.

Thrombocytopenia is typically associated with dengue fever but has also been reported in cases of scrub typhus.^6^,^7^ In this study, we found thrombocytopenia in all three groups. Platelet count was found to be lower in patients with co-infection compared to those with isolated dengue fever as well as scrub typhus. Thrombocytopenia in both infections is believed to be immune-mediated; hence, co-infected patients probably had synergistic immune suppression of platelets leading to lower platelet counts.^8^,^9^ The lowest platelet counts are observed in dengue fever between days 5 and 8.^10^ This study suggests that a trend towards earlier fall and attainment of nadir in platelet count in Dengue patients might be an indicator of co-infection with scrub typhus in endemic areas. Conversely in a patient with scrub typhus, marked thrombocytopenia may be a clue to the presence of co-infection by dengue virus, especially during an outbreak. The study by Ahmad et al. had estimated the time to recovery of thrombocytopenia, unlike this study where we sought to determine time to attain lowest platelet count.

Elevation of transaminases has also been described in both dengue fever and scrub typhus.^11^,^12^ In both these tropical illnesses aspartate aminotransferase (AST) is found to be elevated more than alanine aminotransferase (ALT), a finding that we noted in this study as well. In the case of co-infection, both AST and ALT were significantly higher compared to dengue or scrub typhus alone. A low serum albumin was more likely in co-infection compared to isolated dengue (p<0.001) and may, therefore, serve as a predictor of associated scrub typhus in dengue patients. Hypoalbuminemia and leukocytosis have been reported as independent predictors of severe scrub typhus.^13^ In our study patients with co-infection had hypoalbuminemia, but none of them fulfilled the criteria for severe scrub typhus. Similar to findings of the present study, elevated transaminases (AST > ALT), bilirubin and alkaline phosphatase were also noted in the study by Ahmed et al. and a case of dengue – scrub co-infection reported by Iqbal et al.^4^,^5^

Prolonged activated partial thromboplastin time (aPTT) is a well-known association in case of dengue fever. The combination of prolonged aPTT, normal prothrombin time, leukopenia, thrombocytopenia and elevated transaminases is highly predictive of acute dengue infection.^14^ In this study, we found isolated aPTT prolongation in co-infection and isolated dengue fever. We observed that patients with co–infection had significant prolongation of aPTT compared to isolated scrub typhus but not dengue fever. Hence, a prolonged aPTT in patients with scrub typhus needs to be evaluated to rule out a co-existing dengue infection.

Among outcome-related variables, co-infection led to a significantly longer stay in hospital than dengue fever. Although we could not identify the reasons for the excess days spent in hospital by patients with co-infection, there is no doubt that this would increase the cost of treatment, a major concern in developing countries. Moreover, the patients with co-infection of our study spent relatively more days in the hospital compared to the similar co-infection cohort described by Ahmad et al. A well designed prospective study may provide vital information regarding these aspects. We did not observe any mortality among our co-infection cohort unlike the study from North India where one death was noted.

Appropriate and timely laboratory investigations help to resolve a suspicion of mixed infection. NS1 antigen and IgM ELISA for dengue and IgM for scrub typhus with high cutoff values detected simultaneously, are strong indicators of concomitant infection. NS1 is presumed to be detectable from the first day and remains positive till about a week to ten days. Hence, it is taken as a diagnostic marker for ongoing infection. A combination of IgM and NS1 has a greater predictive value for current dengue infection. They have high sensitivity and specificity.^15^ Detection of these parameters indicates recent or ongoing infection. An IgG detection alone must not be relied upon for ongoing infection. Cross-reactivity between other flavivirus infections and a non-specific response must be borne in mind while interpreting IgM and IgG levels. A panel of tests, with high specificity, have a better chance to differentiate an acute infection from a reactionary response. IgM detection in scrub typhus depends on the type of antigen used to capture the antibodies. Several authors have documented variable results. The manufacturers of ELISA kits have used antigens which detect IgM at the earliest, around 3–4 days.^16^ The ideal confirmatory tests like PCR (which has high specificity almost 100% for scrub typhus, but low sensitivity) for the detection of specific genomes may not be feasible in resource-poor settings. In the present study, past infection by either one was ruled out, as both Dengue NS1 and IgM antibodies for scrub typhus were detected simultaneously.

Apart from the differences in length of hospital stay and the time to attain nadir platelet counts, this study found a general trend towards comparable outcomes between co-infection and mono-infections. One explanation is that neither the patients with mono-infection nor those with co-infection had features to suggest severe disease (dengue shock syndrome or severe scrub typhus). A second possible explanation is that similar to suppression of viral replication of HIV by scrub typhus co-infection,^17^ co-infection could have also compromised dengue viral load. However, this has not been proven so far by in vitro studies. Lastly, prompt initiation of antibiotics (either empirically before test results or soon after results) in co-infected patients could have altered the course of illness nullifying the anticipated summative effects of a co-infection.

The major limitation of this study is its retrospective nature. Second, the number of cases of co-infection is small. Nonetheless, we have been able to describe a co-infection that may easily be overlooked in tropics because of very similar clinical and laboratory features and possibly masked by the use of empiric antibiotics. It is also likely that many cases of co-infection could have been missed as we only included cases of scrub typhus diagnosed by IgM ELISA. However, this strict inclusion based on an extremely sensitive test for scrub typhus is also the strength of our study.^18^ Although the reason for testing IgM scrub antibodies in most of the cases appeared to be the persistence of fever, the deficiency in records precluded further analysis. Moreover, other factors like prior use of antibiotics, dengue serotype and severity of scrub typhus could have confounded our results, thus limiting the applicability of our findings to differentiate co – infection from mono-infection.

## Conclusion

Dengue and scrub typhus co-infection may be an under-recognized combination of concurrent infections in the tropics, particularly India. Features such as tachycardia, low systolic blood pressure, normal leukocyte counts, early drop in platelet counts and hypoalbuminemia in a case of dengue fever should alert the clinician to order additional investigations that include a sensitive test for scrub typhus, either an IgM detection or PCR if available. Shorter time to attain nadir thrombocytopenia may be another important feature that suggests co-infection and may necessitate ordering tests to detect the same. Timely detection and treatment of concurrent infection with *O. tsutsugamushi* may have significant implications as it is likely to reduce the length of hospital stay and in turn the cost of therapy. Larger prospective studies are needed to estimate the exact magnitude and impact of this co-infection.

## Figures and Tables

**Table 1:** Clinical characteristics of patients with dengue-scrub typhus co-infection (n=6).

Characteristics	Dengue (n=18)	Scrub (n=18)	Dengue and Scrub (n=6)	p value
Age*	25.0 (19.8 – 31.3)	25.0 (19.8 – 31.3)	42.5 (33.5 – 58.3)	0.02
Duration of fever*	4.0 (3.8 – 5.2)	10.0 (9.0 – 10.3)	5.5 (3.8 – 6.3)	< 0.001
**Headache**	11 (61.1)	10 (55.6)	4 (66.7)	1.00
**Arthralgia**	3 (16.7)	1 (5.6)	3 (50.0)	0.05
**Rash**	1 (5.6)	1 (5.6)	2 (33.3)	0.15
**Eschar**	0 (0.0)	10 (55.6)	2 (33.3)	0.001
**Lymphadenopathy**	1 (5.6)	4 (22.2)	2 (33.3)	0.15
**Hepatosplenomegaly**	0 (0.0)	4 (22.2)	1 (16.7)	0.11

* Median (interquartile range). All other parameters were presented as number (%).

**Table 2:** Comparison of laboratory findings in patients with dengue-scrub typhus co-infection, those with isolated dengue fever and those with isolated scrub typhus.

Parameter	Dengue (n=18)	Scrub (n=18)	Dengue and Scrub (n=6)	p value
**Hemoglobin (g/dL)**	15.7 ± 1.3	11.0 ± 1.5	10.6 ± 1.2	< 0.001
**Total leucocyte count (×10^9^/L)**	3.9 ± 0.7	11.6± 2.9	8.8± 1.0	< 0.001
**Lowest platelet count (×10^9^/L)**	44.5 ± 28.5	63.8 ± 22.4	21.7 ± 11.5	0.002
**Urea (mg/dL)**	18.2± 7.4	41.0± 17.3	31.8± 7.2	< 0.001
Creatinine* **(mg/dL)**	0.7 (0.6 – 0.8)	0.8 (0.5 – 1.1)	1.1 (0.9 – 1.2)	0.07
**Sodium (mEq/L)**	136.6± 2.6	140.7 ± 10.6	134.7± 6.9	0.14
**Potassium (mEq/L)**	4.2 ± 0.4	3.9 ± 0.6	3.9 ± 0.7	0.28
**Total bilirubin (mg/dL)**	0.5 ± 0.3	1.6 ± 0.3	1.6 ± 0.2	< 0.001
Direct bilirubin*(mg/dL)	0.1 (0.1 – 0.3)	0.4 (0.2 –.05)	0.5 (0.4 – 0.6)	0.002
AST*(U/L)	117.5 (65.5 – 184.3)	95.5 (64.8 – 127.5)	177.5 (121.8 – 250.8)	0.05
ALT*(U/L)	56.5 (32.8 – 75.0)	51.0 (42.5 – 89.5)	99.5 (73.9 – 168.3)	0.02
Alkaline phosphatase* **(U/L)**	69.5 (52.0 – 85.0)	100.0 (84.5 – 112.3)	108.4 (101.0 – 126.3)	0.01
**Albumin (g/dL)**	3.9 ± 0.3	2.9 ± 0.3	3.0 ± .4	< 0.001
Prothrombin time* **(seconds)**	12.9 (11.2 – 13.6)	12.0 (12.0 – 12.3)	12.5 (12.0 – 13.3)	0.50
**aPTT (seconds)**	51.0 ± 8.9	44.8 ± 7.2	57.7 ± 6.0	0.003

* Median (interquartile ranges). All other parameters in mean ± standard deviation.

**Table 3:** Comparison of outcome variables between patients with dengue-scrub typhus co-infection and isolated dengue fever.

Parameter	Dengue (n=18)	Scrub (n=18)	Dengue and Scrub (n=6)	p value
**Hospital stay in days** (mean ± standard deviation)	7.8 ± 1.0	9.6 ± 2.1	10.7 ± 1.2	0.001
**Time to nadir thrombocytopenia from onset of fever in days (**Median (inter-quartile range))	5.0 (5.0 – 6.0)	4.5 (4.0 – 5.0)	3.0 (2.0 – 4.3)	0.001
